# 638. When Size Matters: Pediatric Weights as Part of the Pre-hospital Report

**DOI:** 10.1093/jbcr/irag033.475

**Published:** 2026-04-06

**Authors:** Debbie Boyett, Carey K Lamphier, Jasmin Mercedes, Steve Elmgren, Joshua A Ridgely, Ashley Webb, Pamela Vanderberg, Alexandra K Monroe, Laura S Johnson, Lauren B Nosanov

**Affiliations:** Grady Memorial Hospital, Atlanta, GA; Grady Memorial Hospital, Atlanta, GA; Grady Memorial Hospital, Atlanta, GA; Grady Memorial Hospital, Atlanta, GA; Emory University School of Medicine, Atlanta, GA; Grady Memorial Hospital, Atlanta, GA; Grady Memorial Hospital, Atlanta, GA; Emory University/Grady Memorial Hospital/Children's Healthcare of Atlanta, Atlanta, GA; Grady Memorial Hospital / Emory School of Medicine / Morehouse School of Medicine, Atlanta, GA; Emory University School of Medicine, Atlanta, GA

## Abstract

**Introduction:**

A major benefit of ambulance transport is the ability for pre-hospital providers to give receiving hospitals advanced notification of details for incoming critical patients. Appropriate care of pediatric patients requires an accurate weight to identify size-specific supplies and anticipate medication dosing in a timely manner. Routine quality improvement (QI) case review at our urban regional burn center identified a pediatric patient whose care could have been optimized had Emergency Department (ED) and Burn providers been able to anticipate the patient’s weight and prepare accordingly. Recognizing that age is not an ideal substitute for pediatric weight, we implemented a quality improvement initiative to obtain pre-hospital weights for all incoming pediatric burn patients.

**Methods:**

The Burn Program collaborated with the hospital Communications Office in January 2024 to request an estimated weight for all pediatric burn patients (age < 18 years) from pre-hospital providers when taking reports. This weight value was then transmitted with the remainder of patient information via the existing paging system. The Burn QI team began reviewing all page details for patients presenting via ambulance to the ED to determine if a pre-hospital weight was obtained and transmitted. An audit log was created at this time to monitor compliance with weight reporting, and weekly meetings were held between the QI team and Communications Office to discuss opportunities for process improvement.

**Results:**

On average, our center saw approximately 15 pediatric patients (volumes ranging 8-20) transported to the ED by prehospital providers as burn activations per month. Following the inception of the “pedi weight” text field value, our tracking identified a steady increase in compliance (Fig. 1), with rates reaching 100% at the time of this review. Our routine burn QI process has identified no further cases where a lack of estimated patient weight prior to arrival was identified as an opportunity for improvement.

**Conclusions:**

Evaluation of our process improvement intervention demonstrates successful adoption of a protocol to obtain and disseminate prehospital pediatric burn weight estimates. This has corresponded with an improvement in associated patient outcomes, however, our ability to identify the method by which these estimates were obtained and our ability to confirm accuracy is limited. While our hospital uses the Broselow System for pediatric weight estimation, this method is not standardized for all ambulance services in the area. We are currently working to compare collected prehospital estimated weights to scale weights obtained in the ED to gauge accuracy.

**Applicability of Research to Practice:**

Next steps include working to establish reliable and uniform tools for prehospital provider utilization. We also aim to further assess downstream clinical effects to fully appreciate impact on our overall pediatric readiness.

**Funding for the study:**

N/A.

